# Supplementary material for: Modeling glioblastoma heterogeneity as a dynamic network of cell states
Source: Mol Syst Biol. 2021 Sep 16;17(9):e10105. doi: 10.15252/msb.202010105 (PMC8444284; doi:10.15252/msb.202010105)
Supplement: Supplementary file 6 — Source Data for Figure 5 [file MSB-17-e10105-s004.zip › Figure5A_sourcedata/GSEA_3017/hallmarks_stateF.GseaPreranked.1621934628066/HALLMARK_DNA_REPAIR.html]

Details for gene set HALLMARK\_DNA\_REPAIR[GSEA]

|  || Dataset | state33017 |
| Phenotype | NoPhenotypeAvailable |
| Upregulated in class | na\_neg |
| GeneSet | HALLMARK\_DNA\_REPAIR |
| Enrichment Score (ES) | -0.293156 |
| Normalized Enrichment Score (NES) | -1.4142144 |
| Nominal p-value | 0.10344828 |
| FDR q-value | 0.13191707 |
| FWER p-Value | 0.815 |
Table: GSEA Results Summary

  

Fig 1: Enrichment plot: HALLMARK\_DNA\_REPAIR      
 Profile of the Running ES Score & Positions of GeneSet Members on the Rank Ordered List

  

| PROBE | GENE SYMBOL | GENE\_TITLE | RANK IN GENE LIST | RANK METRIC SCORE | RUNNING ES | CORE ENRICHMENT || 1 | RRM2B |  |  | 22 | 1.148 | 0.0644 | No |
| 2 | SDCBP |  |  | 27 | 1.054 | 0.1308 | No |
| 3 | DDB2 |  |  | 143 | 0.682 | 0.1231 | No |
| 4 | AK1 |  |  | 399 | 0.470 | 0.0387 | No |
| 5 | NME3 |  |  | 424 | 0.461 | 0.0578 | No |
| 6 | XPC |  |  | 425 | 0.461 | 0.0876 | No |
| 7 | REV3L |  |  | 999 | 0.310 | -0.1503 | No |
| 8 | BCAM |  |  | 1049 | 0.300 | -0.1530 | No |
| 9 | TK2 |  |  | 1115 | 0.290 | -0.1635 | No |
| 10 | PDE4B |  |  | 1404 | 0.256 | -0.2766 | Yes |
| 11 | POLD4 |  |  | 1430 | 0.255 | -0.2714 | Yes |
| 12 | RFC5 |  |  | 1473 | -0.250 | -0.2741 | Yes |
| 13 | RPA2 |  |  | 1490 | -0.256 | -0.2647 | Yes |
| 14 | ADRM1 |  |  | 1497 | -0.258 | -0.2507 | Yes |
| 15 | ERCC1 |  |  | 1530 | -0.267 | -0.2479 | Yes |
| 16 | COX17 |  |  | 1589 | -0.284 | -0.2556 | Yes |
| 17 | POLA2 |  |  | 1622 | -0.291 | -0.2512 | Yes |
| 18 | NELFE |  |  | 1631 | -0.294 | -0.2358 | Yes |
| 19 | PRIM1 |  |  | 1703 | -0.320 | -0.2470 | Yes |
| 20 | RAD51 |  |  | 1723 | -0.326 | -0.2345 | Yes |
| 21 | LIG1 |  |  | 1797 | -0.361 | -0.2440 | Yes |
| 22 | RFC4 |  |  | 1887 | -0.413 | -0.2573 | Yes |
| 23 | POLD3 |  |  | 1918 | -0.436 | -0.2426 | Yes |
| 24 | RFC2 |  |  | 1954 | -0.457 | -0.2288 | Yes |
| 25 | ARL6IP1 |  |  | 2001 | -0.494 | -0.2175 | Yes |
| 26 | FEN1 |  |  | 2018 | -0.511 | -0.1917 | Yes |
| 27 | NME4 |  |  | 2029 | -0.520 | -0.1626 | Yes |
| 28 | ZWINT |  |  | 2069 | -0.577 | -0.1428 | Yes |
| 29 | SSRP1 |  |  | 2091 | -0.630 | -0.1114 | Yes |
| 30 | RFC3 |  |  | 2098 | -0.642 | -0.0726 | Yes |
| 31 | ALYREF |  |  | 2111 | -0.677 | -0.0342 | Yes |
| 32 | RPA3 |  |  | 2112 | -0.678 | 0.0097 | Yes |
| 33 | DUT |  |  | 2155 | -0.824 | 0.0441 | Yes |
Table: GSEA details [plain text format]

  

Fig 2: HALLMARK\_DNA\_REPAIR: Random ES distribution      
 Gene set null distribution of ES for **HALLMARK\_DNA\_REPAIR**
